# HIPK2 in the physiology of nervous system and its implications in neurological disorders

**DOI:** 10.1016/j.bbamcr.2023.119465

**Published:** 2023-06

**Authors:** F. Sardina, A. Conte, S. Paladino, G.M. Pierantoni, C. Rinaldo

**Affiliations:** a Institute of Molecular Biology and Pathology (IBPM), Consiglio Nazionale delle Ricerche (CNR), c/o Sapienza University, Rome, Italy; b Department of Molecular Medicine and Medical Biotechnology, University of Naples Federico II, 80131 Naples, Italy

**Keywords:** HIPK2, Kinase, Neurological disorders, Signaling, Molecular medicine

## Abstract

HIPK2 is an evolutionary conserved serine/threonine kinase with multifunctional roles in stress response, embryonic development and pathological conditions, such as cancer and fibrosis. The heterogeneity of its interactors and targets makes HIPK2 activity strongly dependent on the cellular context, and allows it to modulate multiple signaling pathways, ultimately regulating cell fate and proliferation. HIPK2 is highly expressed in the central and peripheral nervous systems, and its genetic ablation causes neurological defects in mice. Moreover, HIPK2 is involved in processes, such as endoplasmic reticulum stress response and protein aggregate accumulation, and pathways, including TGF-β and BMP signaling, that are crucial in the pathogenesis of neurological disorders. Here, we review the data about the role of HIPK2 in neuronal development, survival, and homeostasis, highlighting the implications in the pathogenesis of neurological disorders, and pointing out HIPK2 potentiality as therapeutic target and diagnostic or prognostic marker.

## Highlights

• The kinase HIPK2 is highly expressed in the central and peripheral nervous system.
• HIPK2 modulates several pathways in a cell- and environment-dependent manner.
• HIPK2 plays a critical role in neuronal homeostasis and development.
• HIPK2 is implicated in neurodegeneration by regulating protein turnover and cell death.
• Altered HIPK2 functions are found in different neurological disorders.

## 1 Introduction

Homeodomain-interacting protein kinase 2 (HIPK2) is the most studied member of the HIPK family, which consists of four members in vertebrates. HIPK1-3, identified for their ability to interact with homeodomain transcription factors [1], share a high degree of homology, whereas HIPK4 is the most divergent member of the family [2].

HIPK2 is a stress-responsive kinase acting downstream several signal transduction pathways, in response to a growing list of various stimuli, such as DNA damage, oxidative stress, endoplasmic reticulum (ER) stress, hypoxia, and glucose/nutrient deprivation. Accordingly, alterations of this kinase have been reported in pathological conditions such as cancer and fibrosis. These alterations are quite heterogeneous, and appear to be cell- and context-dependent. For instance, HIPK2 expression is decreased in some tumors, including thyroid and breast carcinomas [3], [4], whereas it is increased in others, like cervical cancer [5] and pilocytic astrocytoma [6]. These discrepancies have been explained considering that the activity of HIPK2 strongly depends on its post-translational modifications, intracellular localization, and molecular interactions, all features that can be differently regulated in diverse cell types.

A growing body of evidence also indicates a role for HIPK2 in neuronal physiology and pathology. While the role of HIPK2 in cancer is well described elsewhere [for example in the following reviews [7], [8], [9], [10]], here we review the molecular and functional interplay among HIPK2 and key neurological signaling pathways, pointing out its physiological role in nervous system development and homeostasis, and its potential involvement in neurological disorders, including neurodegenerative diseases.

### 1.1 HIPK2 isoforms and kinase domain

**Fig. 1:**
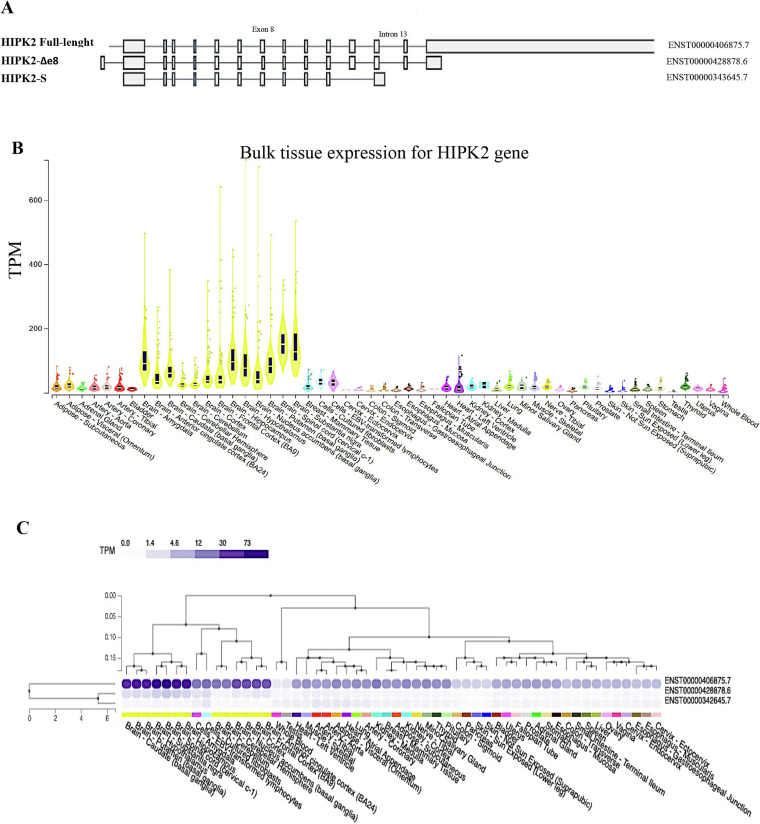
HIPK2 expression in human tissues. A) Scheme of HIPK2 isoforms modified from the Genome-Tissue expression (GTEx; https://gtexportal.org). B) Violin plots for HIPK2 gene from GTEx project RNA-seq data. Transcript levels per million (TPM) are shown. C) HIPK2 isoform expression from GTEx project RNA-seq data. Brain tissue expression is reported in yellow. (For interpretation of the references to colour in this figure legend, the reader is referred to the web version of this article.)

In humans, distinct alternative spliced variants of *HIPK2* gene (Ensembl gene ID ENSG0000006439.15) have been identified [11], [12], [13] (Fig. 1). The most prevalent form of HIPK2 is encoded by the transcript variant 1 (NCBI Reference: NM_022740.5; Ensembl ID ENST000000406875.7; Fig. 1A), producing the full-length 1198 amino acid protein, containing an N-terminal kinase domain, followed by a homeodomain-interacting domain, a SUMO-Interaction Motif, and additional modules responsible for protein-protein interactions, regulation of subcellular localization (the speckle-retention signal, SRS) and stability (the C-terminus auto-inhibitory domain, AID).

The HIPK2-Δe8 isoform (Ensembl ID ENST00000428878.6; Fig. 1A), generated by the skipping of the exon 8, lacks only 27 amino acids which are required for the binding to the E3 ubiquitin ligase Siah-1. Differently from the full-length protein, HIPK2-Δe8 is resistant to proteasome digestion [11], [12].

The HIPK2 short spliced variant (Ensembl ID ENST00000342645.7; Fig. 1A), named HIPK2-S, lacks the last two exons and retains the intron 13, generating a protein product of 1018 amino acids, lacking large part of the AID domain and characterized by 30 extra amino acids at the C-terminus [13]. In other vertebrates the existence of spliced variants is unknown. It will be interesting to investigate whether they are a distinguishing characteristic of human beings.

In this review, we will use the term HIPK2 to describe general characteristics and activities of the kinase, whereas specific features of the alternative spliced variants will be reported, when available.

Like the other HIPK proteins, HIPK2 belongs to the CMGC [Cyclin-dependent kinases (CDK), Mitogen-activated protein kinases, Glycogen synthase kinases and CDK-like kinases] family of protein kinases, and shares the same kinome tree branch with the dual-specificity tyrosine phosphorylation-regulated kinases (DYRKs) [14], [15]. CMGC kinases have a common activation mechanism based on the phosphorylation of a conserved tyrosine residue in the activation loop, that is Y361 for HIPK2, inside a STY motif. This modification occurs through *cis*-autophosphorylation during ribosomal biosynthesis, a mechanism ensuring that the newly synthesized molecule is constitutively activated [14], [15], [16]. The kinase domain of HIPK2 contains an important evolutionary conserved lysine residue (K228, by using NM_022740.5 as reference) that is required for its catalytic activity.

Scarce structural information is available for this kinase. Only recently, the crystal structure of HIPK2 kinase domain was obtained [17]. Despite the high similarity with the other CMGC kinases, this domain contains distinguishing features, opening the possibility to develop more selective inhibitors for specific targeting.

### 1.2 HIPK2 regulation and post-translational modifications

HIPK2 expression varies in response to different cellular stimuli. Alternative splicing [11], [12], [13], microRNAs and circular RNAs [18] concur in regulating *HIPK2* mRNA transcription, translation and degradation; whereas post-translational modifications modulate protein stability, activity and localization [7], [8], [19], [20], [21]. A very complex set of post-translational modifications, including phosphorylation, ubiquitylation, SUMOylation, acetylation and caspase cleavage have been reported to affect HIPK2 activity and modulate its tissue-specific and cell-context dependent functions. These post-translational modifications act in a combinatorial manner. Thus, it has been proposed the existence of a modification code, through which HIPK2 responds to stimuli of different types and intensity, and can integrate various signaling cues. As a consequence, HIPK2 differentially coordinates diverse signaling pathways [22].

For instance, in basal conditions, HIPK2 protein levels are generally very low, mostly because of its constant degradation achieved by the activity of different E3-ubiquitin ligases, such as Siah1, Siah2, MDM2 and WSB-1; whereas, in response to genotoxic stress its levels increase because of protein stabilization, and several post-translational modifications concur to modulate its functions. Deciphering how different regulatory mechanisms allow the fine-tuning of HIPK2 levels and activity in different spatio-temporal conditions is still an open issue. Further investigations in this field could open the possibility of fully understanding HIPK2 specific functions, and attempting specific targeting.

### 1.3 Expression level and tissue distribution of HIPK2

*HIPK2* gene is widely expressed in different human tissues (Fig. 1B). Compared to other tissues, the central nervous system (CNS) shows very high levels of HIPK2 expression (Fig. 1B), suggesting the existence of a different type of regulation of this kinase in the neuronal compartments, and a critical role for brain functions. In particular, the full-length HIPK2 transcript shows high expression levels in brain and spinal cord tissues. Similar results were obtained in the same tissues by analyzing the expression of HIPK2-Δe8 isoform [11], [12] (Fig. 1C). HIPK2-S transcript displays a ubiquitous expression pattern, characterized by heterogeneous expression levels [13], and very low expression in brain tissues (Fig. 1C).

Intriguingly, among the different brain areas, full-length and Δe8 isoforms show the lowest expression in the cerebellum, a brain portion in which it has been observed a peculiar timing of expression compared to the other nervous system areas in mice. In fact, while *Hipk2* expression peaks at p1 and then decreases in the hippocampus, cortex and striatum, it increases over time in the cerebellum, reaching a peak at the latest analyzed time-point (p245) [23]. Consistently, the genetic ablation of *Hipk2* is associated with atrophic cerebellar lobules, strong reduction in cerebellar Purkinje neurons and astrogliosis in adult mice [23]. It would be interesting to evaluate whether *HIPK2* mRNA expression levels increase during aging in the cerebellum of human adults as well.

### 1.4 Intracellular distribution in physiological and pathological contexts

HIPK2 intracellular localization is crucial for its activity, determining with which proteins the kinase can interact, and which substrates it can phosphorylate. Under basal conditions, HIPK2 localizes in both nuclear and cytoplasmic compartments, and it displays a dynamic nucleocytoplasmic shuttling, depending on cellular context [24], [25], [26], [27].

HIPK2 is not homogeneously distributed into the nucleus, but it is rather concentrated in nuclear subdomains, nuclear “speckles” (reviewed in [28]), and its SUMO-interacting domain appears essential for this localization [26], [29]. Moreover, it has been reported that HIPK2 localizes at the promyelocytic leukemia nuclear bodies (PML-NBs) and Polycomb Group (PcG) bodies, structures that are critical for genome stability and transcription repression [28].

Differently from the full-length, HIPK2-S shows a diffuse non-speckled nuclear distribution and a diffuse cytoplasmic staining [13]. Unfortunately, no data on HIPK2-Δe8 subcellular localization are available so far.

Overall, it will be important to understand the dynamics of HIPK2 partition in different nuclear domains, as well as the mechanisms regulating its distribution. Stress conditions, like oxidative stress or treatment with apoptosis activators, lead to HIPK2 stabilization and its accumulation into the nucleus, where it can exert its pro-apoptotic functions, including the activation of the tumor suppressor p53 through the phosphorylation of its serine 46 (S46) [30], [31]. Moreover, the non-receptor tyrosine-kinase Src is able to prevent HIPK2-induced p53 phosphorylation by redistributing HIPK2 from the nucleus to the cytoplasm [32]. In general, diminished nuclear localization of HIPK2 has been associated with malignant phenotypes and poor prognosis in human tumors [33]. Altogether, these data indicate that the fine regulation of HIPK2 nuclear distribution is critical for cellular homeostasis. At the same time, a potential physiological role of HIPK2 in the cytoplasm cannot be excluded. Recent findings showing that a significant amount of HIPK2 is present in the cytosol of cortical, hippocampal, striatal and cerebellar neurons of adult mice [23], support this hypothesis, and point out a potential critical role for cytoplasmic HIPK2 in neuronal physiology.

It is intriguing that HIPK2 is well represented in both cytoplasmatic and nuclear compartments, whereas HIPK1 expression is mostly cytoplasmatic and HIPK3 expression is restricted to the nucleus [27]. Thus, it is likely that the appropriate nuclear/cytosolic levels are critical for tuning HIPK2 functions.

In proliferating cells, HIPK2 has been reported to localize at the midbody, the transient organelle-like structure present at the intercellular bridge during cytokinesis, where this kinase is crucial for successful abscission and midbody remnant removal, through autophagy-mediated degradation [34], [35], [36], [37]. The midbody localization appears to be specific for the HIPK2-S isoform [13]. Interestingly, the timing, the positioning and the fate of the midbody are key events during early brain development [38]. Despite the low expression of HIPK2-S in the brain, if HIPK2-regulated midbody fate might affect neurogenesis is an intriguing question that remains to be determined.

A better understanding of the mechanisms regulating HIPK2 localization will be very important in future. At the same time, altered localization of HIPK2, which could prevent proper interaction with its nuclear targets and/or losing its cytosolic functions, is likely to lead to the development of pathological conditions.

Taken together, all these observations suggest that the characterization of the intracellular distribution of the different HIPK2 isoforms is crucial to fully understand its pathophysiological role.

## 2 HIPK2 in neuronal homeostasis and development

### 2.1 HIPK2 in embryogenesis and neurodevelopment: insights from animal model studies

**Fig. 2:**
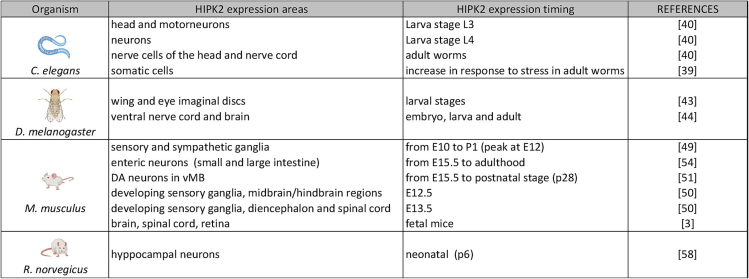
HIPK2 expression in animal models. Schematic summary of HIPK2 expression areas and timing in different organisms, created in part with BioRender.com.

During development and post-natal life, HIPK2 is strongly expressed in multiple neuronal cell types of brain, spinal cord and retina in different organisms (Fig. 1, Fig. 2). Hence, several animal models, including worms, fruit flies, and mice, have been used to study HIPK2 physiological role in the nervous system. *HIPK2* orthologous genes are highly conserved across species. In simple organisms, such as *Drosophila melanogaster* and *Caenorhabditis elegans*, only one homologue of the HIPK family exists.

In *C. elegans*, the single *HIPK2* ortholog, *hpk-1*, appears not essential for development, despite its expression during larval stages (Fig. 2). hpk-1 is involved in lifespan regulation by transcriptionally controlling heat and oxidative stress response, and aging. In particular, hpk-1 regulates key stress-response genes, such as phase I and phase II detoxification enzymes and age-regulated genes, including superoxide dismutase 3 (*sod-3*) and UDP-Glucuronosyl Transferase 9 (*ugt-9*) [39], [40] (Fig. 2).

In Drosophila, *Hipk* exerts many of the same functions described for its vertebrate orthologous [41]. In particular, *Hipk* regulates Notch signaling during eye development, and homeodomain transcriptional factors activity during neurogenesis (Fig. 2) [42]. *Hipk* is expressed in the eyes and wing imaginal discs, and in the brain and ventral nerve cord in embryos, larvae and adults [43], [44]. Wang and co-authors showed that knockdown or overexpression of *Hipk* in neurons produces early adult lethality [45]. To explore the mechanisms underlying this lethality, they analyzed larval neuromuscular junctions and muscle, showing that muscle-specific *Hipk* overexpression negatively impairs the distribution of the cytoskeletal protein Hu-li tai shao (Hts; adducin in mammals) in the muscle. *Hipk*-overexpression also suppresses the expression of two kinases involved in neuromuscular structure/function, such as Calcium-calmodulin protein kinase II (CaMKII) and Partitioning-defective 1 (PAR-1), thus causing a decrease in the phosphorylation levels of the scaffolding protein Discs large (Dlg), which is important for cell junction formation, establishment of cell polarity, and regulation of proliferation. *Hipk* also controls the levels of TBPH, the fly ortholog of TAR DNA-binding protein 43 (TDP-43), associated with the pathogenesis of different neurological diseases. In particular, *Hipk* overexpression strongly decreases the nuclear levels of TBPH.

In mice, Hipk2 promotes cortical neurogenesis, a process occurring from E9 to E16. Hipk2 is expressed in undifferentiated cortical neural progenitor cells from E13.5, when active cortical neuronal differentiation takes place (Fig. 2). The mechanism underlying HIPK2 pro-differentiation role has been, at least in part, elucidated by Ciarapica and co-authors [46]. In combination with the phosphorylation-dependent prolyl isomerase PIN1, Hipk2 promotes differentiation by limiting the anti-neurogenic activity of the Groucho/Transducin-like Enhancer of split 1:Hairy enhancer of split 1 (Gro/TLE1:Hes1) transcriptional repression complex, in a manner dependent on the catalytic activity of both Hipk2 and PIN1. In particular, Hipk2/PIN1 interaction with the Gro/TLE1:Hes1 complex leads to its hyperphosphorylation, associated to a weakening of the binding of Gro/TLE1 to chromatin, with a consequent de-repression of differentiation-promoting markers, such as the microtubule associated protein 2 (*Map2*), RNA-binding Fox-1 Homologue 3 (*Rbfox3*, coding for the protein NeuN) and class III β-tubulin [46]. *Hipk2* genetic ablation results in multiple alterations in mice, revealing the involvement of the kinase in the development and homeostasis of multiple organs and tissues. In particular, *Hipk2*-knock-out (KO) mice present neuronal loss, morphological alterations, and satellitosis throughout the whole CNS; a myopathic phenotype characterized by variable fiber size, mitochondrial proliferation, sarcoplasmic inclusions, morphological alterations at neuromuscular junction; and a cardiac phenotype characterized by fibrosis and cardiomyocyte hypertrophy [47]. Moreover, the double KO of *Hipk2* and High-Mobility Group A1 (*Hmga1*, a gene coding for a non-histone chromatin protein, previously identified as HIPK2 interactor and substrate) causes perinatal death from respiratory failure, associated with impaired lung development and reduction in surfactant proteins, as well as reduced expression of thyroid differentiation markers [48], suggesting that HIPK2 is also involved in the development of endoderm-derived organs.

### 2.2 Role in nervous system development and maturation

During nervous system development, the neuron number progressively increases. When neurogenesis is complete (E13.5 in mice), the programmed cell death (PCD) starts, and excess neurons undergo apoptosis in response to different signal transduction pathways. The complex array of neurotrophic cues that regulate PCD might induce divergent outcomes (death versus survival promotion), depending on spatio-temporal distribution and levels of neurotrophin factors, their receptors, and other adaptors, such as Nerve Growth Factor (NGF), Trk receptors, and specific signaling factors, including TGF-β, BMP, and NF-κB pathway members. HIPK2 is one of the crucial players which regulates these divergent outcomes in a context-dependent manner. In analogy with its proapoptotic effect in DNA-damage response, HIPK2 promotes apoptosis in response to stress induced by inadequate amounts of neurotrophic levels in sensory and sympathetic neurons. In vivo analyses have shown that HIPK2 expression peaks during PCD [49] (Fig. 2), and *Hipk2* genetic ablation results in a sensory neuron increase in mice [50]. Consistently, experiments performed in neuronal cultures revealed that HIPK2 overexpression induces caspase-dependent apoptosis, that might be counteracted by expressing anti-apoptotic factors, such as BCL-2 or BCL-w, whereas HIPK2 downregulation reduces apoptosis in response to neurotrophic withdrawal [49]. Mechanistically, HIPK2 interacts with the homeodomain transcription factor Brn3a/Pou4F1, and transcriptionally suppresses the expression of pro-survival genes, such as *TrkA* and *Bcl-xL*, in sensory neurons [50]. Moreover, it has been observed that *Hipk2* mRNA levels increase upon NGF deprivation in neuron cultures, suggesting that HIPK2 itself is regulated at transcriptional level during the stress response to neurotrophic deprivation.

In other neuronal cell types, HIPK2 plays an opposite role. In fact, it has a pro-survival activity in the dopaminergic (DA) neurons of the ventral midbrain (vMB) and enteric nervous system (ENS), which control voluntary motor movement and intestinal motility, respectively. These DA neurons express HIPK2 after the completion of the early stage of neurogenesis (starting from E15.5; Fig. 2), and their survival, development and maturation critically depends on TGF-β/BMP-SMAD pathways. HIPK2 physically interacts with the SMAD transcription factors, which are downstream mediators of TGF-β and BMP signaling. Following TGF-β or BMP interaction with their receptors, SMAD factors are phosphorylated and translocate to the nucleus, regulating the expression of their target genes. SMAD2/3 factors are canonical TGF-β effectors, whereas SMAD1/5/8 commonly act upon BMP/receptor engagement. It has been reported that HIPK2/SMAD2 and HIPK2/SMAD3 interactions can promote the transcriptional activity of several pro-survival TGF-β genes in immortalized human cells, by using a luciferase reporter under the control of SMAD binding elements [51]. Consistently, DA neurons explanted from *Hipk2*-KO mice are not able to respond to pro-survival signals upon TGF-β activation [49]. In agreement with findings obtained in neuronal cultures, and consistently with HIPK2 expression pattern, Zhang and co-authors observed significantly high levels of apoptotic death of DA neurons in *Hipk2*-KO mice, while DA neurogenesis seems to be unaffected [51]. These phenotypes resemble those of TGF-β2 and SMAD3-deficient mice [52], [53], supporting the idea that HIPK2 is a key factor for TGF-β signaling after neurogenesis completion.

Through a different mechanism, HIPK2 plays a critical role in the postnatal survival and development of DA enteric neurons and glial cells [54]. In early postnatal life, mice lacking Hipk2 show several gastrointestinal defects, including gut motility impairment associated with a high postnatal reduction of DA enteric neurons, and an increase in glia density. The *Hipk2*-KO enteric neurons show maturation arrest, reduced synaptic density, and an increased number of autophagosomes in the axons and cell bodies, associated to high levels of the microtubule-associated protein-1 light chain 3 (LC3), suggestive of increased autophagy. This phenotype is evident already at P0 and persists in adult mice, indicating that the enteric neurons require *Hipk2* expression also postnatally, likely for synaptogenesis and maturation [54]. Consistently, Hipk2 is detectable in very few glia at P0 with a progressive increase from P16 to P32, during neuronal development; while in neurons, *Hipk2* expression is scattered at E15.5 and P0, with an extensive presence along the wall of small and large intestine at E15.5 to adulthood (Fig. 2). The mechanism underlying the DA enteric neuron reduction in *Hipk2*-KO mice is still unclear, but it seems to involve autophagy activation rather than conventional apoptotic cell death [54]. To support this hypothesis, HIPK2 has been recently reported as a critical player in the regulation of autophagy in primary mouse hepatocytes and in HeLa cells [37], [55].

Maturation and survival of DA enteric neurons are controlled by the BMP/SMAD signaling pathway. The loss of *Hipk2* leads to an increase of SMAD1/5/8 phosphorylation in enteric neurons, suggesting that it causes an excessive BMP signaling in these cells. A functional interaction between Hipk2 and the BMP/SMAD pathway has been demonstrated also in other cellular contexts. For instance, the physical interaction between Hipk2 and SMAD1 negatively regulates the transcription of BMP reporter genes in murine immortalized C2C12 muscle cells [56]. Even if further studies are needed, it is possible to speculate that the ENS development might be regulated by Hipk2 via modulation of the BMP/SMAD signaling pathway.

**Fig. 3:**
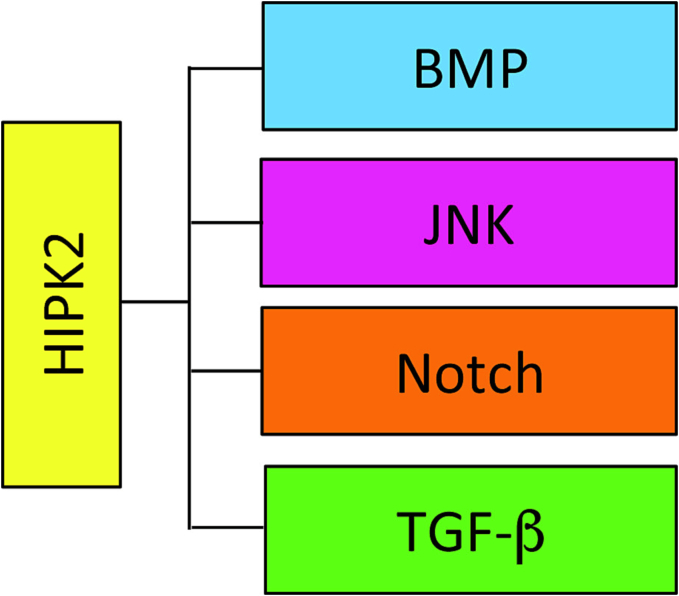
HIPK2 pathways in nervous system. Scheme summarizing the main signaling pathways affected by HIPK2 in neuronal cells.

In vMB DA neurons, it has been reported that Hipk2 regulates the transcription of the two ionotropic glutamate NMDA receptor subunits, named GluN2A and GluN2C, that are critical for neuron communication. This regulation occurs via JNK/c-JUN signaling in the substantia nigra (SN), in the cerebral cortex and in the spinal cord. The loss of Hipk2 leads to a decrease of this signaling, promoting an upregulation of GluN2C and GluN2A levels resulting in an increase in the GluN2A/GluN2B ratio, and in a persistent activation of the pro-survival ERK-CREB pathway in early postnatal brain. These changes stimulate the upregulation of several anti-death genes, enhancing the cell death resistance of *Hipk2*-KO embryonic DA neurons to mitochondrial toxins, like carbonyl cyanide *m*-chlorophenyl hydrazone (CCCP) [57]. While apparently contradictory, the ambivalent role of HIPK2 in DA neurons represents an example of how the effects of this kinase are strongly dependent not only on the cellular context, but also on the environmental conditions. In fact, on one hand, *Hipk2* genetic ablation increases cell death of DA neurons during physiological mouse embryonic development, and, on the other hand, it makes the same neurons resistant to apoptosis in the presence of mitochondrial toxins. In general, the effects of HIPK2 on cell survival depends on the type and intensity of the stimuli that trigger its activation, and on the pathophysiological context, ranging from pro-apoptotic to pro-survival by regulating different pathways (Fig. 3) [10].

### 2.3 HIPK2 and synaptic maturation and transmission

Given the role of Hipk2 in controlling the levels of A and C subunit of NMDA receptors, it has been suggested that Hipk2 has also a role in the switch from slow GluN2B-containing NMDA receptors to faster GluN2A-containing NMDA receptors during early postnatal mouse development [57], a switch that is critical for synaptic maturation, circuit development, and associative learning. Further evidence of a link between synapses and Hipk2 emerges from its involvement in the long-term synaptic toxicity of neonatal exposure to sevoflurane, one of the most widely used anesthetics [58]. In rat hippocampal neurons, Hipk2 and JNK/c-Jun were found to be upregulated shortly after neonatal exposure to sevoflurane, and this upregulation persists in adults. Sevoflurane exposure does not affect the numbers of hippocampal neurons, but results in a decrease of excitatory synapse number, and synaptic protein levels associated with memory impairment. Notably, these phenotypes can be rescued by using HIPK2 or JNK antagonists, indicating HIPK2-JNK/c-Jun signaling as a potential target to reduce sevoflurane toxicity.

## 3 HIPK2 in neurological disorders

### 3.1 Alzheimer's disease (AD)

AD is a primary progressive neurodegenerative disease, characterized by neuronal cell death caused by an aberrant metabolism of the amyloid precursor protein (APP), resulting in the production and deposition of amyloid beta (Aβ) peptides (Aβ 1-40 and Aβ 1-42 isoforms), that are the main constituents of the extracellular amyloid plaques, a hallmark of this disease. A link between p53 and the perturbation of APP metabolism has been demonstrated in several studies. In particular, the altered conformational state of p53, independent of point mutations, has been reported to cause a dysfunctional response to stressors in tissues from AD patients. HIPK2 is one of the main p53 regulators, controlling its activity and conformational stability. Indeed, in the absence of HIPK2, p53 acquires a misfolded conformation, losing DNA binding and transcriptional activities, that depend on metallothioneins (MT) expression and Zn^2+^ availability. The binding of Zn^2+^ ions to the p53 DNA-binding domain is essential for the correct folding and activity of the protein. HIPK2 is able to repress the expression of the metallothionein 2A (MT2A), a protein that chelates Zn^2+^ ions, preventing them from binding p53 and stabilizing its 3D conformation. In the absence of HIPK2, MT2A levels increase, causing p53 misfolding due to the lack of available Zn^2+^ [59].

The effect of HIPK2 on p53 protein stability is perturbed by the Aβ 1–40 peptide in different cell systems [60]. In particular, Aβ peptides cause a down-regulation of the HIPK2 interactor Zyxin, resulting in an increased proteasomal degradation of HIPK2. The decrease of HIPK2 levels leads to its disappearance from the MT2A target promoter. The consequential induction of MT2A is responsible for p53 misfolding and inhibition of p53 transcriptional activity [61], making cells unable to properly activate an apoptotic program. This mechanism suggests that soluble Aβ peptides can be responsible for important modulatory effects at cellular level, even before triggering the amyloidogenic cascade.

### 3.2 Amyotrophic lateral sclerosis (ALS)

ALS is an adult-onset progressive disease characterized by neurodegeneration of upper and lower motor neurons. ALS can be either sporadic (sALS) or familial (fALS), and four genes (superoxide dismutase 1 (*SOD1)*, TAR DNA binding protein (*TARDBP)*, fused in sarcoma (*FUS),* and *C9ORF72*) are causally linked to the pathogenesis, and account for the majority of fALS cases. Mutations of these fALS genes cause dysfunctions of RNA metabolism and protein homeostasis, often associated with impairment of the ubiquitin-proteasome pathway and ER stress [60]. The potential involvement of HIPK2 in the pathogenesis of ALS has initially been hypothesized by Lee and co-authors, who showed that Hipk2 can link ER stress to cell death via the IRE1a-ASK1-JNK pathway in murine embryonic fibroblasts (MEFs), cortical neurons, and spinal cord motor neurons [62]. ER stress is an important hallmark of neurodegenerative diseases, caused by the accumulation of abnormally folded proteins that activates highly conserved signal transduction pathways, collectively known as the unfolded protein response (UPR). Under ER stress, UPR upregulates genes modulating protein folding and degradation, which can reduce the excess of unfolded proteins from the ER. However, prolonged ER stress can activate irreversible signaling pathways that lead to cell death. It has been demonstrated that the fALS-causative SOD1 G93A mutation induces ER stress, and activates Hipk2 by phosphorylating its highly conserved serine and threonine residues (S359/T360) within the activation loop, causing Hipk2-JNK association that leads to apoptosis activation in motor neurons. Consistently, *Hipk2* genetic ablation delays disease onset, reduces cell death in spinal motor neurons, mitigates glial pathology, and improves survival in the SOD1-G93A fALS mice model.

Another significant evidence of the involvement of HIPK2 in the pathogenesis of ALS is represented by its association to TDP-43, a ubiquitously expressed nuclear DNA/RNA-binding protein that is involved in several RNA processing events, including splicing, transcription, and translation. Interestingly, even if *TARDBP* gene mutations account only for a small percentage of fALS cases, TDP-43 expression levels and subcellular localization are altered in almost all ALS patients, with TDP-43 toxic cytoplasmic aggregates (“TDP-43 proteinopathy”) observed in approximately 97 % of all ALS patients. Remarkably, HIPK2 activation positively correlates with TDP-43 proteinopathy in sALS, *C9ORF72* fALS, and in mice expressing a cytoplasmic TDP-43 mutant (i.e., NEFH-tTA/tetO-hTDP-43ΔNLS). Consistently, the inhibition of HIPK2 kinase activity by using a compound, named A64, protects motor neurons from TDP-43 cytotoxicity [62].

Hipk2 is also able to promote the ubiquitin-dependent degradation of Wip1, a phosphatase that dephosphorylates several key signaling proteins involved in stress response, including ATM, CHK2, p53, p38 MAPK, MDM2, and the p65 component of the NF-κB complex, all of which play critical roles in apoptosis, cell cycle, and DNA repair. Wip1 is downregulated in SOD1-G93A mice, and the lack of its activity is associated with a hyper-activation of stress signaling pathways that ultimately leads to apoptotic cell death in ALS motor neurons [63].

Based on these data suggesting that HIPK2 may be an important player in the pathogenesis of ALS, HIPK2 kinase inhibitors have been proposed as potential therapeutic compounds for ALS [64].

### 3.3 Parkinson disease (PD)

PD, the second most common neurodegenerative disorder after AD, results from a complex interplay of genetic and environmental factors. The pathological hallmark of PD is the progressive loss of DA in the substantia nigra pars compacta (SNpc) and the presence of protein aggregates (Lewy bodies), mostly composed of α-synuclein, in surviving neurons. The resultant dopamine deficiency within the basal ganglia leads to the classical parkinsonian motor symptoms [65].

Because of its role in DA neuron survival [51], HIPK2 has been proposed as a potential player in PD pathogenesis. Consistently with this idea, *HIPK2* mRNA was found to be downregulated in the prefrontal cortex of PD patients compared to healthy controls [51]. In order to find potential HIPK2 inhibitors and investigate their effects in neuronal cells, Wang and co-authors tested for their ability to bind HIPK2, several compounds extracted from *Cannabis sativa*, a plant whose consumption represents a putative factor in longevity and health of aboriginals living in the Guangxi province of China, one of the five largest longevity regions in the world [66]. Using molecular modeling and molecular dynamics simulations, they found that the (Z)-methyl p-hydroxycinnamate (ZMHC) binds HIPK2 with high affinity. Interestingly, ZMHC treatment attenuates the apoptosis induced by a mitochondrial toxin in SH-SY5Y DA neuronal cell model [66]. The authors speculate that this effect is due to HIPK2 inhibition, but, even if they show that ZMHC treatment decreases the pro-apototic p53 S46 phosphorylation, they do not provide a formal demonstration of the ability of this compound to inhibit the HIPK2 kinase activity. However, their data suggested that the lack of HIPK2 function may be associated with protection from mitochondrial toxin activity in neuronal cells [66].

Similarly to other neurodegenerative diseases, mitochondrial dysfunction and accumulation of damaged mitochondria may concur to PD pathogenesis. Damaged mitochondria are normally degraded via the autophagosome pathway, and a key mediator of this process (called mitophagy) is the protein Parkin, a E3 ubiquitin ligase that was initially identified in patients affected by familial PD. Under basal conditions, Parkin is mainly cytosolic, but is quickly recruited to the outer mitochondrial membrane to promote mitophagy upon treatment with mitochondrial uncouplers, like CCCP [67]. Moreover, Parkin decreases the expression levels of parkin interacting substrate (PARIS or ZNF746), a transcriptional repressor that inhibits the expression of target genes that are critical for mitochondrial functions, such as the peroxisome proliferator-activated receptor-g coactivator-1a (PGC-1a). Zhang and co-authors have recently demonstrated that *Hipk2* genetic ablation affects the Parkin turnover, thus exerting a protective effect in mouse neurons treated with mitochondrial uncouplers. In fact, mitochondria of *Hipk2*-KO MEFs are more resistant to CCCP-induced changes in mitochondrial membrane potentials and integrity, compared to their wild-type counterpart. This resistance to toxin effects is associated with an increase in cytoplasmic and mitochondrial levels of Parkin, and in the expression of its downstream effector PGC-1a. Consistently, the authors showed that HIPK2 promotes proteasome-dependent Parkin degradation, in a kinase activity-dependent manner [68].

Taken together, these data suggest that HIPK2 may be involved in the pathogenesis of PD, but the mechanisms and the relevance of this potential involvement remain to be clarified. Moreover, the importance of HIPK2 in DA neuron maturation and survival emerged from KO mice characterization [51], corroborates the hypothesis that this kinase may be involved also in neuropsychiatric disorders characterized by DA circuitry dysfunction, such as schizophrenia and addiction. Hence, further investigations of the mechanisms of action of HIPK2 in DA neurons will be very important because they may open the possibility to envisage strategies to improve the efficacy of DA neuron circuitry, with potential therapeutic implications in neuropsychiatric and neurodegenerative diseases.

### 3.4 Hereditary spastic paraplegia (HSP)

The HSPs are a heterogeneous group of rare genetic diseases, characterized by progressive weakness and spasticity of the lower extremities, due to degeneration of corticospinal neurons [69]. The most common type of HSP is due to dominant mutations of the *SPG4* gene, encoding spastin, a microtubule severing protein, that controls crucial processes such as cytokinesis, axonal transport and inter-organelle trafficking. *SPG4* mutations lead to pathogenesis mainly by a haploinsufficiency mechanism. To date, there is no effective therapy, but several evidence indicates that spastin elevating approaches might be a viable therapeutic strategy. Recently, it has been demonstrated that HIPK2 phosphorylates spastin at S268 [70]. This modification inhibits spastin K48-poly-ubiquitylation at K554 and prevents its proteasomal degradation in a neddylation-dependent manner [71]. Notably, overexpression of HIPK2 or inhibition of neddylation causes an increase in spastin levels associated with a rescue of pathological defects in SPG4 neuronal models [71]. This evidence provides the proof of principle that HIPK2/spastin axis could represent a promising therapeutic target for spastin elevating treatments in HSP.

### 3.5 Lafora disease

Lafora disease is a progressive fatal autosomal recessive form of myoclonus epilepsy, caused by loss-of-function mutations of *EPM2A* or *NHLRC1* genes, encoding the glycogen phosphatase laforin and the E3 ubiquitin ligase malin, respectively [72]. Aberrant glycogen aggregates, named Lafora bodies, are a hallmark of this disease, and the drivers of its pathogenesis. Laforin and malin show a cellular protective role, acting in proteolytic processes, and glycogen metabolism. Loss of laforin or malin increases HIPK2 expression levels, with a consequent increase of p53 proapoptotic activity and cell death [73]. Specularly, the overexpression of laforin or malin confers protection against HIPK2-mediated cell death, by targeting HIPK2 to the cytoplasmic compartment. These findings suggest that the activation of HIPK2 may be one of the causes of the massive cell death observed in Lafora disease. Therefore, HIPK2-p53 apoptotic pathway could be evaluated as a new possible therapeutic target for this disorder.

### 3.6 ENS pathologies

Intestinal motility is strongly affected by enteric DA neuron defects, and abnormalities in ENS are frequently observed in patients affected by neurodegenerative diseases, such as PD and AD [74]. Considering the importance of BMP/Hipk2 signaling in ENS DA neuron homeostasis [56], it is possible that its regulation could be therapeutically exploited to treat intestinal dysmotility in enteric neuropathies. However, further studies are needed to fully elucidate the molecular mechanisms of action of BMP/HIPK2 signaling in the ENS, and to verify whether significant therapeutic benefits can be achieved targeting it in enteric neuropathies.

### 3.7 Cerebellar ataxia (CA)

Ataxia is a neurological sign consisting of lack of voluntary coordination of muscle movements, that can include gait and eye movement abnormalities, and speech changes. CA is due to dysfunction of the parts of the nervous system that coordinate movement, such as the cerebellum, or its connections. Many conditions can cause CA, including alcohol misuse, stroke, tumor, brain degeneration, multiple sclerosis, certain medications and genetic disorders. A potential role of Hipk2 in cerebellar homeostasis and ataxia emerged from the behavioral and morphological analysis of *Hipk2*-KO mice. In fact, the genetic ablation of *Hipk2* results in a loss of Purkinje neurons in the cerebellum of adult mice, which is characterized by astrogliosis, atrophic lobules, and an average size that is overall smaller than that of its wild-type counterpart. At cellular and molecular level, these morphological alterations are associated to an ubiquitinated protein accumulation and apoptosis. Consistently, the *Hipk2*-KO mice also display behavioral anomalies that are typically associated with cerebellar deficits. In particular, they present dystonia characterized by the clasping of hind limbs when suspended by their tails, consistent failure to finish the tandem walk, poor motor coordination, and reduced response to novelty [23]. Moreover, *HIPK2* is one of the genes whose expression has been found to be altered in peripheral blood mononuclear cells isolated from patients affected by Friedrich's ataxia [75], further suggesting *HIPK2* as a novel gene involved in ataxia-like cerebellar disorders.

### 3.8 Rett syndrome (RTT) and CDKL5 deficiency disorder (CDD)

RTT is a severe neurological disorder, which represents the most common genetic cause of severe mental retardation in girls worldwide [76]. The classical form of RTT arises from mutations in the methyl-CpG-binding protein 2 gene (*MeCP2*). The MeCP2 protein is bound and phosphorylated by the cyclin-dependent kinase-like 5 (CDKL5), whose mutations cause CDD, an early onset neurodevelopmental X-linked syndrome, characterized by intractable epilepsy, infantile spasms, and cognitive disabilities [77].

Among the major MeCP2 phosphorylation sites, S421 and S80 modulate MeCP2 chromatin activities, and have been shown to be relevant for neuronal functions. S421 is phosphorylated through a CaMKII-IV dependent mechanism, whereas S80 is phosphorylated by HIPK2. It has been reported that MeCP2 contributes to HIPK2-mediated apoptosis in a S80 phosphorylation-dependent manner [78]. However, it is unknown whether this function is involved in the pathogenesis of the Rett syndrome. Furthermore, Mecp2 has been reported to have cell type-specific effects in regulating the NMDA receptor subunit composition in the visual cortex, during postnatal development. The identification of Hipk2 as a transcriptional regulator of the GluN2B-to-GluN2A switch adds another possibility to its potential implications in the RTT syndrome [57]. The reduction of GluN2A expression in *Mecp2*-KO mice alleviates the decline in visual function, suggesting that targeting NMDA subunit composition might provide feasible therapeutic approaches for Rett syndrome and HIPK2 may represent a strong candidate. Another level of complexity in the interplay between HIPK2 and MeCP2 comes from the results of a genetic screen for druggable regulators of MeCP2 stability that identified the kinase as one of the candidates that can stabilize MeCP2 protein levels in vivo [79], deserving further investigation.

A link between CDKL5 and HIPK2 comes from the observation that both converge at the midbody during cytokinesis [80]. It has been shown that CDKL5 is required for the localization of HIPK2 at the midbody, where it is necessary for successful cytokinesis through phosphorylation of the extrachromosomal histone H2B at S14 [34]. CDKL5 silencing results in loss of HIPK2 at the midbody, cytokinesis defects and poly-ploidization. These phenotypes are rescued by the overexpression of the phosphomimetic mutant H2B-S14D, indicating that they are caused by the impairment of HIPK2-mediated H2B phosphorylation [80]. The relevance of HIPK2/CDKL5 interaction in neuronal functions and its implications in CDD are not clear yet.

### 3.9 Autism spectrum disorders (ASDs)

The ASDs are persistent disabling neurodevelopmental disorders that result in challenges in social interaction, communication difficulties, and stereotypic behaviors. About 50 % of the total risk to develop ASDs is related to environmental factors [81], including the exposure to chemicals during pregnancy. Indeed, gestational arsenic exposure induces autistic behaviors in rats, as demonstrated by Zhou and co-authors [82]. Interestingly, arsenic exposure causes neuronal apoptosis, and upregulation of HIPK2-p53 pathway in the frontal cortex of these rats, suggesting that this pathway could exert a role in ASDs. Consistently with this idea, a clinical study performed on a small cohort of ASD children and age-matched healthy controls, revealed that HIPK2 and p53 protein levels were higher in the sera of ASD children than in those of the healthy controls. Moreover, the HIPK2/p53 upregulation is associated with higher serum levels of 15 elements, among which arsenic, silicon, strontium, and vanadium [82]. These findings suggest that HIPK2 and p53 could be used as biomarkers to detect the effects of exposures to environmental pollutants on ASD development. However, future large population studies are necessary to determine the sensitivity and specificity of HIPK2 and p53 serum level measurements and their real potential in preventive medicine.

## 4 Conclusions and open questions

Multiple signaling factors known to regulate cell proliferation, death and survival in the pathogenesis of cancer, including p53, PIN1, and protein phosphatase 2A, have been recently associated to neurodegenerative diseases [83]. Acting through similar context- and stage- dependent molecular mechanisms in both oncological and neurological diseases, HIPK2 is one of these factors.

**Fig. 4:**
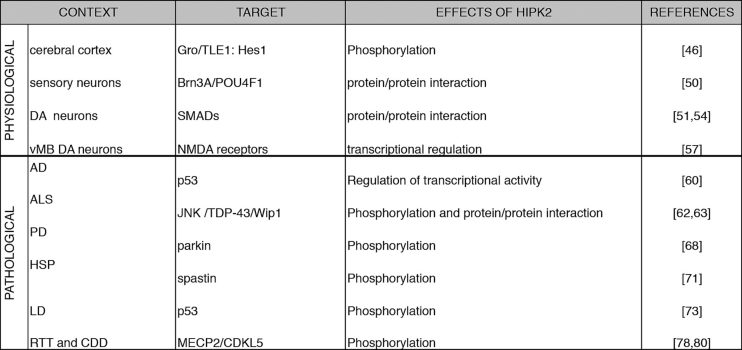
HIPK2 targets. Schematic summary of HIPK2 interactors and substrates in different neuronal physiological and pathological conditions.

**Fig. 5:**
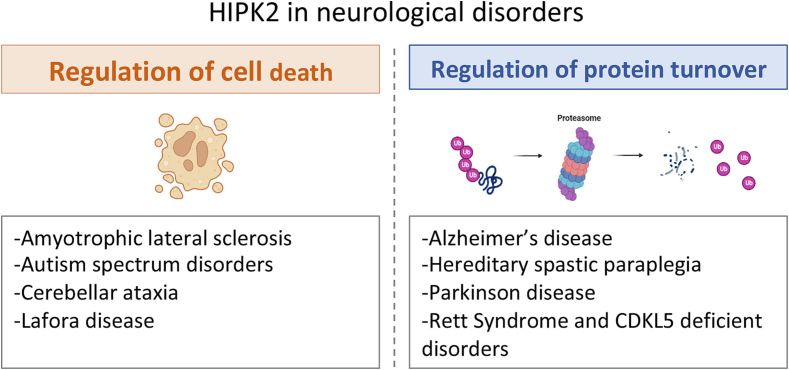
HIPK2 in neurological disorders. Schematic representation of the neurological disorders in which HIPK2 is involved by regulating either cell death or protein turnover, created in part with BioRender.com. Scheme illustrating the main functions of HIPK2 implicated in neurological disorders.

Over the last few years, several reports indicated that HIPK2 plays important physiological functions in the development and the homeostasis of the nervous system (Fig. 2, Fig. 3, Fig. 4). In particular, the data obtained in mouse models revealed that this kinase is required for proper cortical neurogenesis, DA neuron survival, maturation and transmission, and cerebellar functions. At the same time, HIPK2 is involved in several neurological disorders, by impacting crucial cellular processes, such as the regulation of neuronal cell death and protein turnover (Fig. 4, Fig. 5). Its altered expression and/or activity appear to be involved in the pathogenesis of PD, AD, ALS, and CA. Several data suggest that HIPK2 may be a potential biomarker and/or even a new putative therapeutic target for neurological diseases, such as ASDs, Lafora disease, HSP, RETT and ENS pathologies. However, further investigations that could fully elucidate the different mechanisms through which HIPK2 is involved in the pathogenesis of neurological disorders, would be needed to concretely estimate the real translational potential of this factor. The situation is complicated by the fact that HIPK2 activities are highly dependent on the cellular and environmental context, and its role can be protective or detrimental to neuronal and glial cell homeostasis, depending on the circumstances.

To address these issues, the field would benefit from pre-clinical studies performed in animal models to clarify how the modulation of HIPK2 expression and/or activity really impacts the development of neurological disorders, and open the route for clinical studies. A deeper understanding of the molecular mechanisms underlying the diverse neuronal functions of HIPK2 will allow hypothesizing strategies to differentially targeting diverse HIPK2/protein interactions. At the same time, a systematic analysis of large cohorts of patients will be fundamental to evaluate how frequently HIPK2 is deregulated and/or mutated in neurological diseases, and subsequently propose it as a diagnostic or prognostic marker in clinical practice.

## Funding

This study was supported by grants from Telethon #GGP20040 to CR; AFM-Telethon #23786, ASL HSP-France and A.I.Vi.P.S. to FS; and from the Italian Ministry for University and Research (MUR): National Recovery and Resilience Plan (NRRP), Mission 4 Component 2 Investment 1.3 – PE12 Neuroscience (Project code PE0000006) “A multiscale integrated approach to the study of the nervous system in health and disease” (MNESYS) to GMP and SP. The funders had no role in data analysis, decision to publish, or preparation of the manuscript.

## CRediT authorship contribution statement

FS, AC, SP, GMP and CR wrote the manuscript; all authors critically discuss the data in literature. CR and GMP jointly initiated the idea and equally contributed to coordinate the writing and review of the final manuscript. FS and AC prepared the illustrations and equally contributed to the writing. The final version was approved by all the authors.

## Declaration of competing interest

All authors declare that they have no conflicts of interest.

## Data Availability

This review article is based on the information's available on different papers
